# Association between finasteride with subjective memory deficits: a study from the NHANES and FAERS databases

**DOI:** 10.3389/fneur.2025.1616851

**Published:** 2025-06-13

**Authors:** Hao Zhang, Hongqi Ou, Panli Zhao, Xi Luo, Ping Zhang, Hua Huang

**Affiliations:** Department of Pharmacy, Chengdu Seventh People's Hospital (Affiliated Cancer Hospital of Chengdu Medical College), Chengdu, China

**Keywords:** finasteride, memory, NHANES, FAERS, cognitive function

## Abstract

**Background:**

Postmarketing pharmacovigilance data have raised concerns regarding the potential cognitive effects associated with finasteride administration. However, existing epidemiological evidence remains inconclusive, with studies reporting both positive and null associations between finasteride exposure and memory dysfunction. This highlights the need for further comprehensive clinical investigations.

**Objective:**

This investigation employed a comprehensive, multi-source analytical approach to evaluate the potential correlation between finasteride administration and self-reported memory dysfunction, aiming to establish an evidence-based framework for clinical safety evaluation and therapeutic risk–benefit analysis.

**Methods:**

This study incorporated two principal data repositories: the National Health and Nutrition Examination Survey (NHANES, 2001–2018) and the Food and Drug Administration Adverse Event Reporting System (FAERS, 2004–2018). A comprehensive analytical framework was implemented, incorporating descriptive statistics, multivariable logistic regression modeling, and receiver operating characteristic (ROC) curve analysis to examine potential associations between finasteride exposure and cognitive performance metrics.

**Results:**

Multivariable logistic regression analysis of the NHANES dataset, adjusted for demographic variables and lifestyle factors, revealed a significant positive correlation between finasteride exposure and memory impairment risk (adjusted OR = 6.15, 95% CI: 1.62–23.4, *p* = 0.008). Concurrent analysis of the FAERS database identified 6,624 finasteride-related adverse reports, with cognitive dysfunction (*n* = 526) comprising a notable proportion of documented complications.

**Conclusion:**

Convergent evidence from both epidemiological studies and pharmacovigilance surveillance suggests a potential association between finasteride administration and cognitive dysfunction, particularly in memory-related domains. These findings underscore the need for comprehensive risk communication strategies regarding potential neurocognitive adverse effects during clinical consultations and for establishing routine cognitive monitoring protocols for patients undergoing prolonged finasteride therapy.

## 1 Introduction

Finasteride, a selective 5α-reductase inhibitor (5-ARI), is a mainstay treatment for androgenetic alopecia and benign prostatic hyperplasia acting by blocking the enzymatic conversion of testosterone to dihydrotestosterone (DHT) (1). Despite its generally favorable safety profile, concerns have emerged regarding its neuropsychiatric sequelae. This is mechanistically plausible as 5α-reductase inhibition may disrupt neurosteroidogenesis, potentially influencing mood regulation and cognitive processes (2, 3). Memory, a central part of cognitive functioning, encompasses the process of acquisition, storage, and retrieval of information. The early stages of Alzheimer’s disease are usually accompanied by significant deficits in short-term memory, while long-term memory and other cognitive functions are progressively impaired as the disease progresses.

Finasteride has demonstrated a range of therapeutic applications across multiple medical domains. For instance, preclinical investigations have shown its capacity to significantly modulate lipid metabolism and attenuate atherosclerotic progression (4). Furthermore, it has also exhibited substantial neuroprotective properties in Parkinsonian animal models (5, 6). Nevertheless, alongside these broader effects, significant neuropsychiatric and sexual adverse effects have been identified in clinical settings. These manifestations, potentially constituting a distinct clinical entity termed Post-Finasteride Syndrome (PFS), may persist even after treatment cessation, suggesting a unique underlying pathophysiological mechanism (7). Recognizing these safety concerns, the U. S. Food and Drug Administration mandated revisions to finasteride’s prescribing information in 2012 to incorporate warnings regarding depression, erectile dysfunction, male infertility, and impaired semen parameters (8). Contemporary research has increasingly focused on finasteride’s potential neuropsychiatric implications, particularly its association with anxiety spectrum disorders, mood disturbances, and suicidality. Pharmacovigilance analyses, notably those utilizing the FAERS database, have identified persistent sexual dysfunction as a significant treatment-emergent adverse event in androgenetic alopecia management, potentially correlating with increased suicide risk (9). Clinical investigations, such as a longitudinal study by Rahimi-Ardabili et al. (10) involving 128 androgenetic alopecia patients, demonstrated significant elevations in both Beck Depression Inventory (*p* < 0.001) and Hospital Anxiety Depression Scale scores (*p* = 0.005) following 1 mg daily finasteride administration over 12 months. The cognitive impact of 5α-reductase inhibition remains a subject of ongoing investigation, with conflicting evidence. While some Canadian population-based studies initially identified an elevated risk of dementia during early treatment phases (118% first year; 52% second year), long-term use (more than 2 years) showed no significant association (HR 1.06, 95% CI: 0.98–1.14) (11). Further research involving 131 healthy participants has suggested potential correlations between finasteride exposure and memory-related symptoms characteristic of PFS (12). Conversely, evidence from placebo-controlled trials utilizing the Geriatric Depression Scale has failed to demonstrate significant cognitive alterations associated with either finasteride or testosterone enanthate administration (13).

Given the inconsistencies in existing epidemiological evidence regarding finasteride’s cognitive effects, particularly on memory, a comprehensive and multi-source approach is warranted. This investigation employed a comprehensive analytical approach, leveraging both NHANES and FAERS databases to systematically evaluate the association between finasteride exposure and memory function across diverse populations. By analyzing real-world data, this study aims to provide evidence-based insights to inform clinical practice and pharmacovigilance strategies concerning finasteride’s potential impact on subjective cognitive performance.

## 2 Materials and methods

### 2.1 Data sources

This study utilized two primary data sources. The National Health and Nutrition Examination Survey (NHANES), administered by the National Center for Health Statistics (NCHS), is a nationally representative survey employing a complex, multistage probability sampling design to ensure national representativeness. NHANES data collection encompasses comprehensive household interviews for demographic and medical history, complemented by standardized physical examinations and biospecimen collection at mobile examination centers (MECs). All NHANES study protocols received approval from the NCHS Research Ethics Review Board. For the current investigation, NHANES data spanning 2001–2018 were utilized to maintain covariate consistency, with all datasets accessed through official NCHS repositories (14). For pharmacovigilance analysis, OpenVigil 2.1,^1^ a validated pharmacoepidemiological tool extensively utilized in drug safety research (15, 16), served as the primary data mining platform. This platform facilitated the extraction of adverse event reports where finasteride was identified as the primary suspect (PS) medication. The search was conducted for reports between January 1, 2004 and December 31, 2018, specifically using “Finasteride” as the target drug identifier.

### 2.2 Exposure factors

Finasteride exposure in NHANES participants was determined based on self-reported prescription drug use. Participants were asked, “Have you taken or used any prescription drugs in the past month?” Individuals who responded “no” were classified as the control group, while individuals who indicated they had used finasteride were classified as the exposed group. Additionally, the prescription drug questionnaire collected information on the duration of medication use through the question: “How long have you been using or taking (product name)?”

### 2.3 Memory status

Participants in the NHANES database were evaluated for physical functioning during the Mobile Examination Center (MEC) assessment, using the“Experiencing Confusion/Memory” module. Respondents were asked, “Are you limited because you have difficulty remembering or are often confused?” For the purpose of this study, a response of “no” indicates that subjective memory is not significantly affected, while a response of “yes” suggests that subjective memory deficits have a considerable impact on daily life.

### 2.4 Covariates

Based on existing literature and research design requirements (1, 14), this study included the following covariates: age (treated as a continuous variable); gender; race (self-reported their race and were categorized as Mexican American, other Hispanic, non-Hispanic white, non-Hispanic black, and other race); education level (categorized as high school graduate or less, some college, and college graduate or more); body mass index (BMI) [calculated as weight in kilograms divided by height in meters squared (kg/m^2^)]; smoking status (divided into never smokers, defined as having somoked fewer than 100 cigarettes in their lifetime, and smokers defined by having somokef 100 or more cigarettes); caffeine intake; sugar intake; alcohol consumption; diabetes and hypertension (self-reported, based on prior physician diagnosis); and physical activity. Physical activity was assessed based on the question, In a typical week, do you engage in any moderate-intensity exercise, fitness, or recreational activities that result in a slight increase in breathing or heart rate, such as brisk walking, bicycling, swimming, or volleyball, and that last for at least 10 min each time?” The potential confounding effects of antidepressant and benzodiazepine use were excluded from the analysis. Specifically, major representative medications such as fluoxetine, sertraline, citalopram, venlafaxine, alprazolam, and diazepam, among others, were specifically excluded from the analysis.

### 2.5 Statistical analysis

Given the complex multi-stage stratified probability survey design of NHANES, all statistical analyses for this study combined sample weighting, clustering, and stratification. Continuous variables were expressed as means (standard deviation, SD), and categorical variables as counts (percentages). Logistic regression models were used to estimate odds ratios (ORs) and 95% confidence intervals (95% CIs) for the association between finasteride use and memory function. Based on the research (14), the interaction and subgroup analyses were also conducted using logistic regression models stratified by age group, gender, BMI, smoking status, hypertension, physical activity, and diabetes. Sensitivity analyses were performed to assess the robustness of the findings. First, E-values were calculated after performing logistic regression on Model 3. Additionally, diagnostic performance was assessed using receiver operating characteristic (ROC) curves, and the area under the curve (AUC) was calculated to evaluate predictive accuracy (17). Furthermore, adverse events related to memory deficits were mined from the FAERS database to explore the real-world safety profile of finasteride. For FAERS data analysis, the reporting odds ratio (ROR) method was combined with the Bayesian Confidence Propagation Neural Network (BCPNN) method for signal detection (18), and the specific formulas are shown in Tables 1, 2. Each Preferred Term (PT) was screened against the thresholds outlined in Table 2. Both algorithms needed to meet the judgmental criteria before an adverse drug event (ADE) signal was generated. The generation of ADE signals indicates a statistical association with the drug, where a stronger signal implies a stronger correlation. All statistical analyses were conducted using R software version 4.4.0 and Excel, with statistical significance defined as *p* < 0.05.

**Table 1:** Fourfold table for calculation.

	Topotecan	Non-Topotecan
Target AEs	a	c
Non-target AEs	b	d
N = a + b + c + d		

**Table 2:** Formulas and thresholds of ROR and BCPNN.

Method	Formula	Threshold
ROR	ROR= $\frac{a/c}{b/d}$ 95%CI = e^ $\left(\text{lnROR}\pm 1.96\sqrt{1/a+1/b+1/c+1/d}\right)$	a ≥ 3 and 95% CI (lower limit) > 1
BCPNN	IC= $\log_{2}\frac{a(a+b+c+d)}{\left(a+b)(a+c\right)}$ $\gamma =\gamma_{\mathrm{ij}}\frac{\left(N+\alpha )(N+\beta \right)}{\left(a+b+\alpha_{i})(a+c+\beta_{j}\right)}$ $E(\mathrm{IC})=\log_{2}\frac{\left(a+\gamma_{\mathrm{ij}})(N+\alpha )(N+\beta \right)}{\left(N+\gamma )(a+b+\alpha_{i})(a+c+\beta_{j}\right)}$ $V(\mathrm{IC})={\left(\frac{1}{\ln2}\right)}^{2}(\frac{N-\alpha +\gamma -\gamma_{\mathrm{ij}}}{\left(\alpha +\gamma_{\mathrm{ij}})(1+N+\gamma \right)}+\frac{N-a-b+\alpha -\alpha_{i}}{\left(a+b+\alpha_{i})(1+N+\alpha \right)}+\frac{N-a-c+\beta -\beta_{j}}{\left(a+c+\beta_{j})(1+N+\beta \right)})$ $\mathrm{SD}=\sqrt{V(\mathrm{IC})}$ IC025= E(IC)−2SD	IC025 > 0

## 3 Results

### 3.1 Characteristics of participants

**Figure 1:**
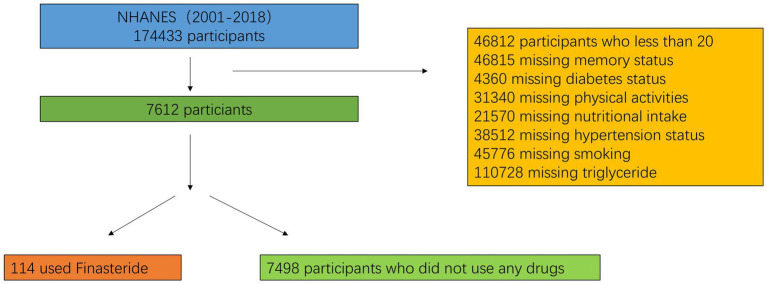
Flowchart of the sample selection from NHANES.

From the 2001–2018 NHANES dataset, 7,612 eligible participants were included after applying stringent exclusion criteria (age <20 years, incomplete memory status data, missing medication records, inadequate nutritional intake information, undefined hypertension/diabetes status, unspecified tobacco use, and insufficient physical activity data). The study population was stratified into two cohorts: finasteride-exposed (*n* = 114) and unexposed (*n* = 7,498) cohorts, with 239 individuals reporting memory-related complaints. The study population had a mean age of 40 years (59% male, 41% female). Individuals reporting memory complaints were significantly older (mean age: 46 years) compared to those without complaints (mean age: 39 years). Racial distribution analysis revealed non-Hispanic whites as the predominant group, comprising 59% of the cohort. Educational attainment analysis revealed that nearly 50% of participants had completed high school or higher education. However, the finasteride-exposed group exhibited a higher proportion of individuals with less than a high school education (50%). The cohort’s mean BMI was 27.5 kg/m^2^, with no significant intergroup differences in sugar, alcohol, or caffeine consumption. Smoking prevalence was marginally higher among memory-complaint individuals (57%) compared to controls (43%). Interestingly, diabetes and hypertension prevalence were lower in the memory-complaint group. While nearly half of the overall cohort reported moderate physical activity, only 32% of memory-complaint individuals met this criterion. Detailed demographic and clinical characteristics are presented in Figure 1 and Table 3.

**Table 3:** Baseline characteristics of the study participants.

Characteristic	N^a^	Overall, *N* = 76,923,916^b^	0, *N* = 74,803,832^b^	1, *N* = 2,120,083^b^	*p*-value^c^
Age	7,612	40 ± (14)	39 ± (14)	46 ± (18)	0.004
Gender	7,612				0.4
Male		4,282 (59%)	4,147 (59%)	135 (55%)	
Female		3,330 (41%)	3,226 (41%)	104 (45%)	
Race	7,612				0.4
Mexican American		1,813 (14%)	1,758 (14%)	55 (12%)	
Other Hispanic		766 (7.3%)	734 (7.3%)	32 (11%)	
Non-Hispanic White		2,711 (59%)	2,619 (60%)	92 (58%)	
Non-Hispanic Black		1,509 (12%)	1,471 (12%)	38 (12%)	
Other Race		813 (8.0%)	791 (8.0%)	22 (8.0%)	
Education	7,612				<0.001
Below high school		3,801 (50%)	3,625 (49%)	176 (74%)	
High school		2,184 (28%)	2,146 (31%)	38 (17%)	
Above high school		1,627 (22%)	1,602 (27%)	25 (12%)	
BMI	7,612	27.5 ± (4.5)	27.5 ± (4.5)	28.3 ± (4.8)	0.062
Triglyceride	7,612	124 ± (114)	123 ± (114)	135 ± (116)	0.3
Sugar	7,612	124 ± (85)	124 ± (85)	121 ± (84)	0.6
Caffeine	7,612	169 ± (223)	169 ± (222)	177 ± (262)	0.4
Alcohol	7,612	14 ± (34)	14 ± (34)	16 ± (40)	0.8
Smoking	7,612				<0.001
Yes		3,158 (43%)	3,034 (42%)	124 (57%)	
No		4,454 (57%)	4,339 (58%)	115 (43%)	
Hypertension	7,612				<0.001
Yes		864 (11%)	813 (11%)	51 (20%)	
No		6,748 (89%)	6,560 (89%)	188 (80%)	
Moderate activites	7,612				<0.001
Yes		3,336 (49%)	3,263 (49%)	73 (32%)	
No		4,276 (51%)	4,110 (51%)	166 (68%)	
Diabetes	7,612				<0.001
Yes		145 (1.2%)	130 (1.1%)	15 (4.1%)	
No		7,467 (99%)	7,243 (99%)	224 (96%)	
Finasteride	7,612				<0.001
No		7,498 (98%)	7,277 (99%)	221 (92%)	
Yes		114 (1.5%)	96 (1.4%)	18 (8.1%)	

a N not Missing (unweighted).

b Mean ± (SD); *n* (unweighted) (%).

c Wilcoxon rank-sum test for complex survey samples; chi-squared test with Rao & Scott’s second-order correction.

### 3.2 Logistic regression results

To investigate the association between finasteride exposure and memory impairment, a multivariable logistic regression analysis was performed using a hierarchical modeling approach with three sequential models constructed to progressively adjust for potential confounding factors. In the unadjusted Model 1, finasteride users demonstrated a 537% increased likelihood of memory impairment compared to non-users (OR = 6.37, 95% CI: 2.85–14.3, *p* < 0.001). While statistically significant, these initial findings may reflect unmeasured confounding effects. Model 2 incorporated demographic covariates including age, gender, race, and educational attainment. After adjustment, the association remained statistically significant albeit attenuated (OR = 6.45, 95% CI: 1.95–21.4, *p* = 0.003), indicating substantial confounding effects of demographic variables. The fully adjusted Model 3 included all potential covariates, revealing a persistent significant association between finasteride use and memory impairment risk (OR = 6.15, 95% CI: 1.62–23.4, *p* = 0.008). These findings suggest that finasteride exposure independently increases the risk of memory-related cognitive dysfunction, even after accounting for a comprehensive set of potential confounding variables, which is shown in Table 4.

**Table 4:** Association between taking finasteride and memory loss.

[OR (95%CI) *p*-value]	Model 1	Model 2	Model 3
Finasteride			
0	Ref	Ref	Ref
1	6.37 (2.85,14.3) < 0.001	6.45 (1.95,21.4) 0.003	6.15 (1.62,23.4) 0.008

Model 1: None adjust; Model 2: Adjust age, gender, race, education; Model 3: Adjust age, gender, race, education, bmi, triglyceride, sugar, caffeine, alcohol, smoking, hypertension, activity, diabetes.

### 3.3 Interaction and subgroup analyses

**Figure 2:**
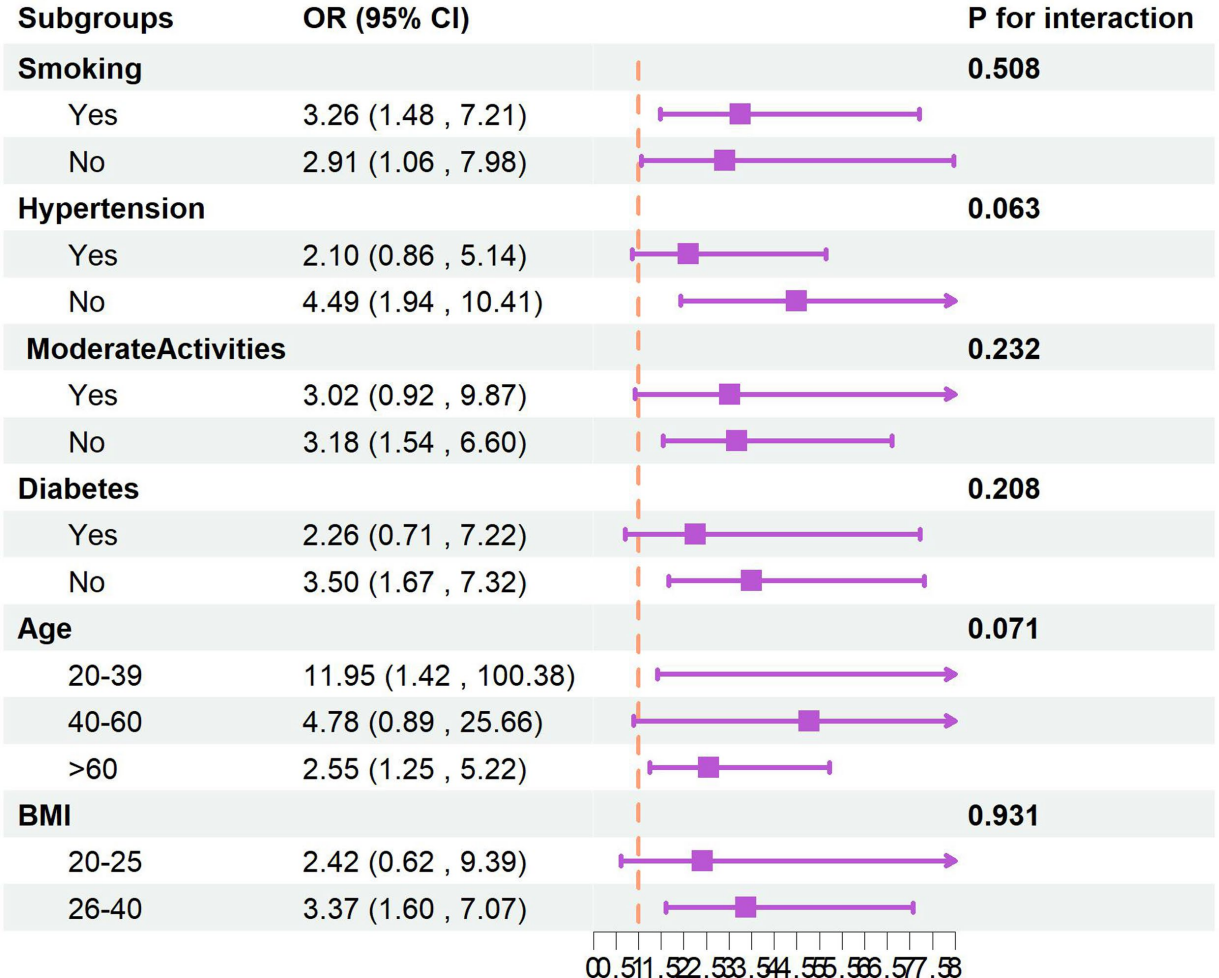
The subgroups analysis.

Stratified analyses incorporating gender, age, BMI, smoking status, hypertension, physical activity levels, and diabetes status revealed no significant effect modification, as evidenced by interaction *p*-values exceeding 0.05 for all covariates. These findings indicate that the observed association between finasteride exposure and memory impairment remains consistent across various demographic and clinical subgroups. Detailed stratification results are presented in Figure 2.

### 3.4 Sensitivity analyses

**Figure 3:**
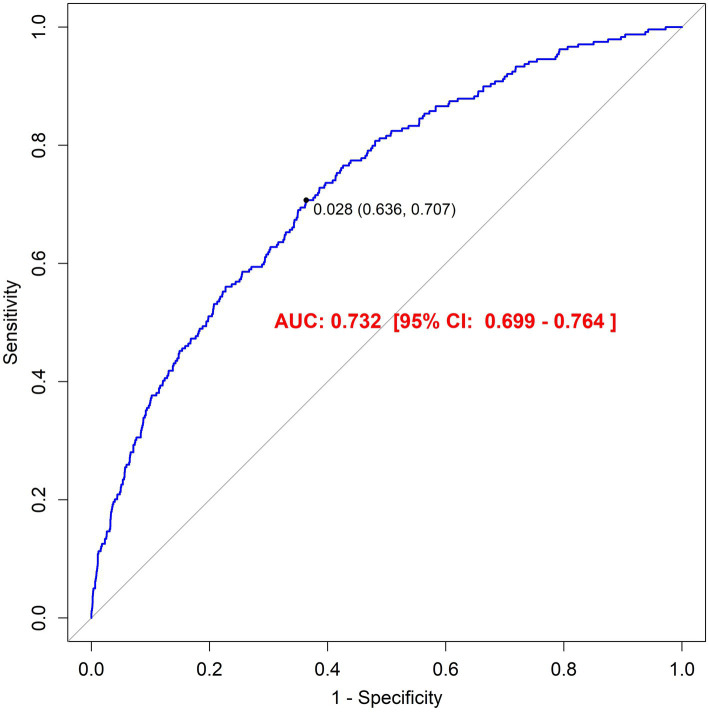
The result of Receiver operator curve (ROC).

Sensitivity analysis using the E-value metric yielded a value of 2.654 (95% CI: 1.37–4.94) following comprehensive covariate adjustment, exceeding conventional thresholds. This indicates that the observed association between finasteride exposure and memory impairment demonstrates robustness against potential unmeasured confounding factors. Furthermore, the model’s discriminative ability was evaluated using receiver operating characteristic (ROC) curve analysis, which yielded an area under the curve (AUC) of 0.732 (95% CI: 0.699–0.764). This AUC value suggests satisfactory predictive accuracy of the model in identifying individuals with memory impairment risk. Detailed results are illustrated in Figure 3.

### 3.5 FAERS database results

**Figure 4:**
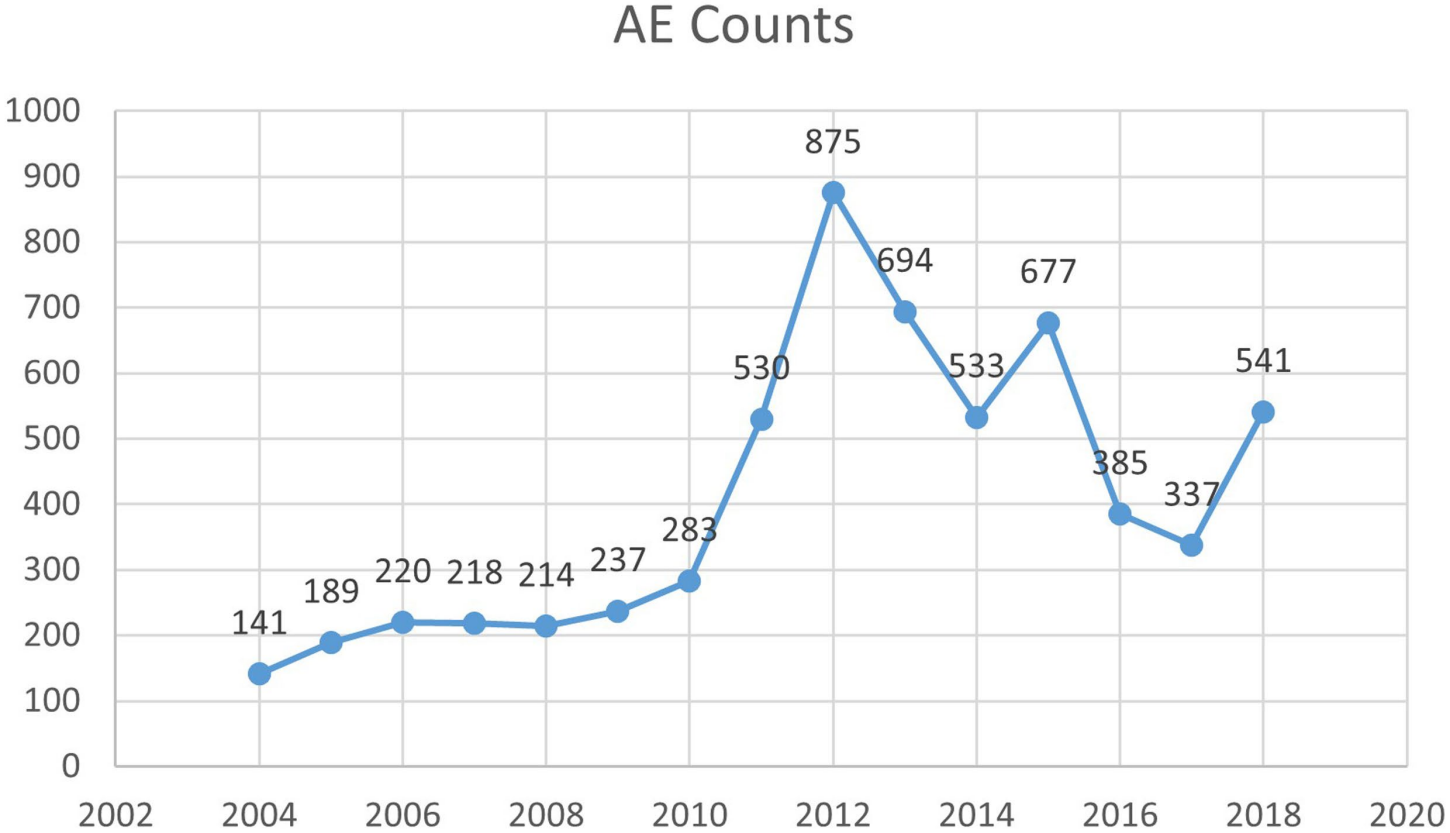
The year of AE counts.

**Figure 5:**
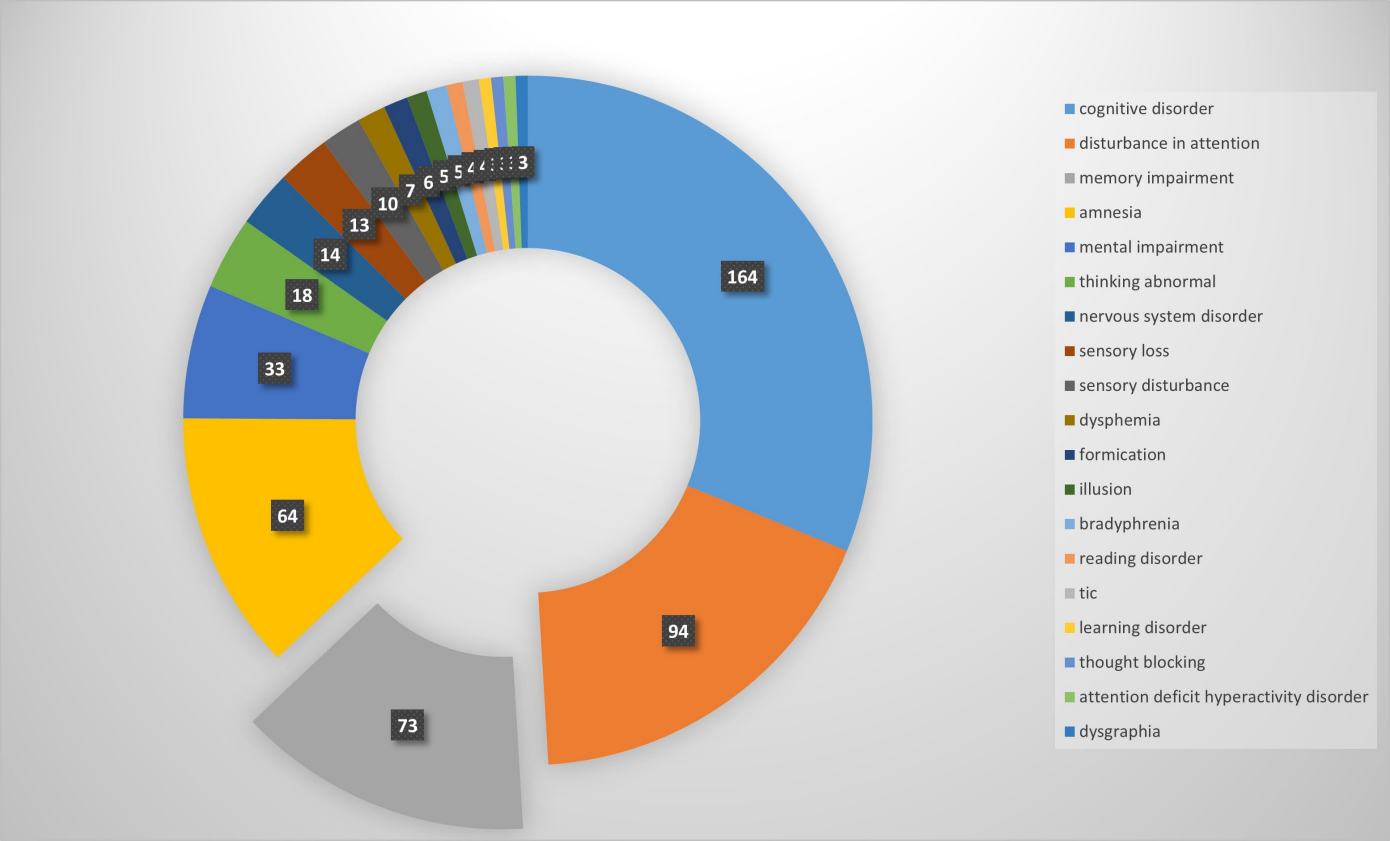
Adverse events count and composition.

**Figure 6:**
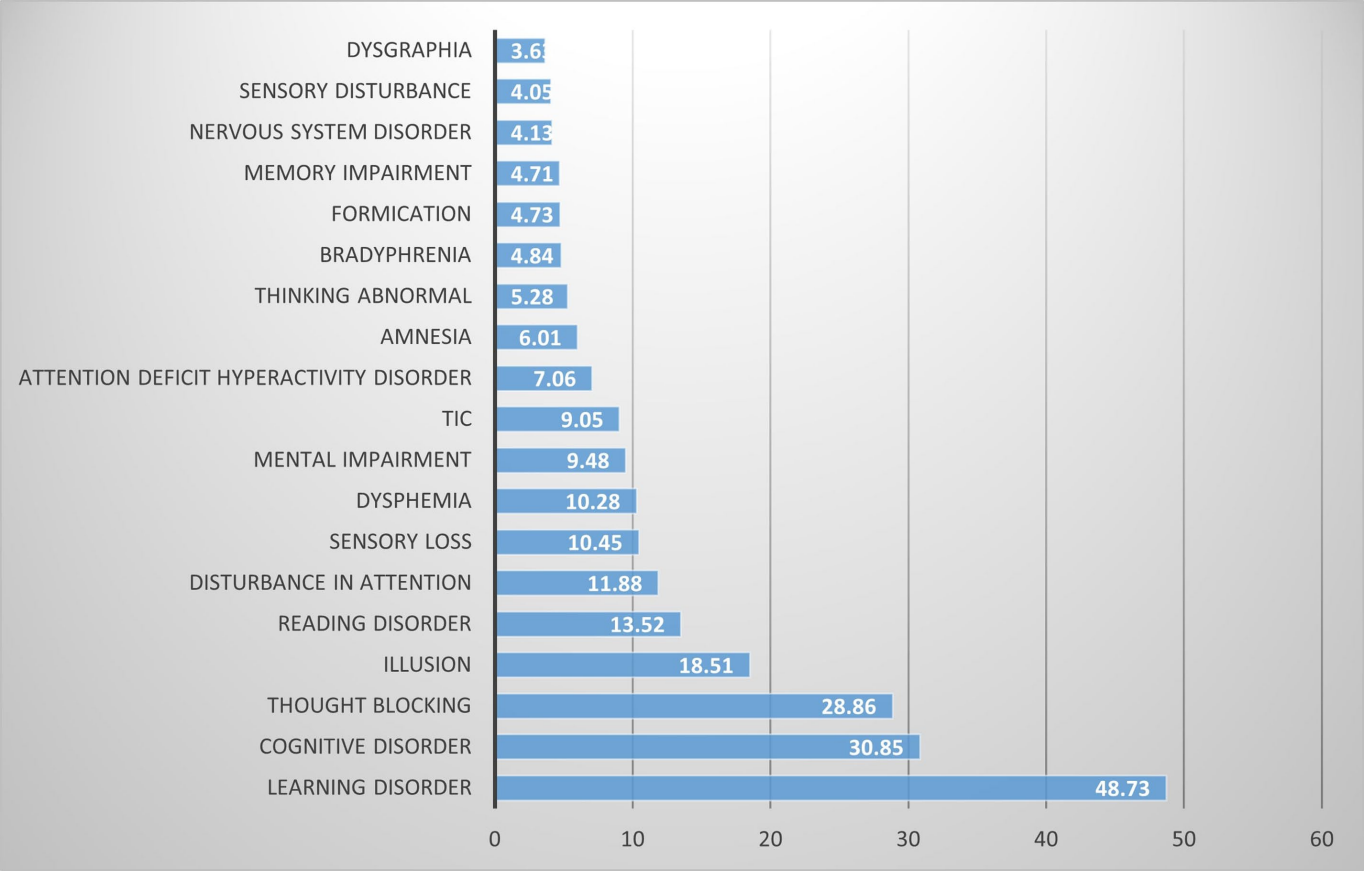
PT of signal intensity.

Our pharmacovigilance analysis, utilizing the FAERS database, systematically identified and quantified adverse event reports where finasteride was designated as the primary suspected agent. The yearly trend of adverse events associated with Finasteride, as illustrated in Figure 4, shows an upward trajectory after 2010, with a peak in 2012. This observed trend may correlate with the global strengthening of pharmacovigilance during that period. The analysis specifically revealed 526 documented cases of memory-related dysfunction, with temporal distribution patterns illustrated in Figure 5. These findings suggest a potential association between finasteride exposure and cognitive deterioration. To evaluate the strength of this association, we employed the Reporting Odds Ratio (ROR) metric with corresponding 95% Confidence Intervals (CIs), as depicted in Figure 6 and Table 5. Notably, the most significant ROR values were observed for learning disorders (ROR = 48.73, 95% CI: 15.36–154.53), cognitive dysfunction (ROR = 30.85, 95% CI: 26.28–36.22), and thought process disturbances (ROR = 28.86, 95% CI: 9.18–90.72). These high ROR values indicate substantial associations between finasteride use and specific neurocognitive impairments in the FAERS database.

**Table 5:** The PT of signal intensity.

PT	ROR	95%CI High	95%CI Low
Learning disorder	48.73	154.53	15.36
Cognitive disorder	30.85	36.22	26.28
Thought blocking	28.86	90.72	9.18
Illusion	18.51	44.81	7.65
Reading disorder	13.52	36.26	5.05
Disturbance in attention	11.88	14.62	9.66
Sensory loss	10.45	18.06	6.04
Dysphemia	10.28	21.65	4.88
Mental impairment	9.48	13.38	6.71
Tic	9.05	24.22	3.38
Attention deficit hyperactivity Disorder	7.06	21.99	2.27
Amnesia	6.01	7.71	4.69
Thinking abnormal	5.28	8.40	3.31
Bradyphrenia	4.84	11.66	2.01
Formication	4.73	10.57	2.12
Memory impairment	4.71	5.95	3.73
Nervous system disorder	4.13	6.98	2.44
Sensory disturbance	4.05	7.54	2.17
Dysgraphia	3.63	11.30	1.17

## 4 Discussion

This comprehensive investigation leveraged the extensive NHANES database (2001–2018) to systematically examine potential associations between finasteride exposure and subjective memory performance. Our multivariable analyses, incorporating adjustments for demographic factors, lifestyle variables (including dietary patterns, substance use), comorbid conditions (such as hypertension and diabetes), and physical activity levels, revealed a significantly elevated risk of memory impairment among finasteride users compared to non-finasteride users. This association maintained statistical significance despite comprehensive adjustment for potential confounders. While interaction analyses and subgroup stratifications yielded non-significant *p*-values (*p* > 0.05) for covariate effects, suggesting limited moderating influences, sensitivity analyses provided robust support for the finasteride-memory relationship through substantial E-values. These findings indicate that unmeasured confounders are unlikely to substantially alter the observed associations. Furthermore, receiver operating characteristic (ROC) curve analysis demonstrated excellent model discrimination, validating the analytical framework’s reliability. Complementary pharmacovigilance analysis utilizing FAERS real-world data identified documented cases of memory-related adverse events following finasteride use. Although representing a minority of reports, these findings suggest potential cognitive risks in susceptible populations, offering valuable insights for clinical decision-making and patient counseling. Collectively, this study provides substantive evidence regarding finasteride’s cognitive effects while highlighting the importance of comprehensive drug safety surveillance through large-scale data analytics.

### 4.1 Neuroactive steroids

Currently, only a limited number of studies have investigated the association between finasteride use and cognitive impairment, with a greater focus on its adverse effects related to depression and anxiety. Finasteride’s mechanism of action, the inhibition of 5α-reductase, directly impacts the synthesis of neucoactive steroids. These steroids, as critical regulators of nervous system function, play pivotal roles in emotional regulation, behavioral performance, reproductive function, and cognitive processes, while also exhibiting neuroprotective properties in models of neurological injury and degenerative diseases (19). Among these, pregnenolone, testosterone, and progesterone are particularly important in the regulation of the nervous system (20). Progesterone, for instance, can directly or indirectly activate signaling pathways, such as the ERK/MAPK and PI3K/Akt pathways, through the modulation of brain-derived neurotrophic factor (BDNF), thereby regulating long-term potentiation (LTP), which is the synaptic substrate for learning and memory (21, 22). Zhang et al. (23) explored the ameliorative effect of translocator protein (TSPO) overexpression on a lipopolysaccharide (LPS)-induced cognitive impairment model in mice. They bilaterally injected GV287 viral vectors mediating TSPO overexpression into the CA1 region of the mouse hippocampus, while establishing a finasteride intervention group as a control. The results demonstrated that TSPO overexpression significantly ameliorated cognitive deficits in the model mice by promoting the biosynthesis of progesterone and isoprogesterone, an effect not observed in the finasteride intervention group (23). Karademir et al. (24) measured serum allopregnenolone levels in 41 patients with bipolar disorder and 40 healthy controls, alongside conducting psychiatric assessments on all participants. Their findings revealed lower serum levels of allopregnenolone and progesterone in patients with bipolar disorder, with serum allopregnenolone levels progressively decreasing as the duration of illness increased. Furthermore, neurocognitive test performance positively correlated with allopregnenolone levels across all participants (24). However, a laboratory study indicated that ovariectomy in aged rats did not impair spatial memory but rather enhanced it, with progesterone counteracting the beneficial effects of ovariectomy (25). Clinical studies on finasteride’s cognitive effects have yielded mixed results. Vaughan et al. (26) categorized older men (65–83 years) without cognitive deficits, who had baseline total testosterone levels below 350 ng/dL, into three groups: 200 mg of testosterone every 2 weeks, 200 mg of testosterone every 2 weeks plus 5 mg/d of finasteride, and placebo. Minimal differences were observed among the three groups in short-term (4-month) or long-term (36-month) analyses of various cognitive performance assessments (26). Conversely, a study by Garcia-Argibay et al. (27) in a Swedish population of men aged 50–90 years demonstrated an elevated risk of all-cause dementia in both finasteride and dutasteride users (finasteride: HR 1.22, 95% CI 1.17–1.28; dutasteride: HR 1.10, 95% CI 1.01–1.20). A similar trend was observed for Alzheimer’s disease risk (finasteride: HR 1.20, 95% CI 1.10–1.31; dutasteride: HR 1.28, 95% CI 1.09–1.50). However, it is important to note that the strength of this association diminished with longer duration of medication and was no longer statistically significant after more than 4 years of continuous exposure (27).

### 4.2 Dendritic spines and synaptic plasticity

Research indicates that synaptic plasticity may mediate the acquisition, consolidation, and retention of memories. Dendritic spines, as prominent and critical components, likely play an important role in immediate memory formation and long-term storage (28). Experimental studies have investigated the impact of finasteride on these neuronal structures. For instance, a study involving 6-month-old male wild-type and 3xTg-AD mice, administered intraperitoneal injection of finasteride (50 mg/kg/d) for 20 days, revealed impaired object recognition memories. Short-term memory deficits were observed only in the 3xTg-AD model, while long-term memory impairment occurred in both wild-type and 3xTg-AD models. Histological analysis further demonstrated that finasteride significantly reduced dendritic branching complexity and dendritic spine density of pyramidal neurons in the CA3 region of the hippocampus in male 3xTg-AD mice. It was also notable that hippocampal amyloid *β* deposition levels were significantly higher in female 3xTg-AD mice compared to male control and finasteride-treated groups (29). These findings collectively raise the speculation that reduced hippocampal dendritic spine density may contribute to the cognitive and psychological symptoms observed in patients with finasteride syndrome (30).

### 4.3 Cholinergic system

Studies suggest that 5α-reductase-mediated neurosteroids like tetrahydroprogesterone or pregnanol sulfate may play a role in regulating cholinergic function (31, 32). Acetylcholinesterase activity, a key marker of the cholinergic system, has been shown to be reduced in patients with Alzheimer’s disease and mild cognitive impairment (33). Ahire et al. (34) trained 2-month-old male Wistar rats to perform a partially baited radial arm maze task, followed by administration of either hydroxypropyl-*β*-cyclodextrin (HPβCD) vehicle or finasteride intervention for 7 days each. Behavioral tests revealed that the finasteride intervention group exhibited significant spatial memory deficits in the radial arm maze task, accompanied by reduced social interaction behaviors. Further biochemical analyses demonstrated that finasteride significantly reduced acetylcholinesterase activity in the frontal cortex, hippocampus, and septum (34). However, some researchers have observed that finasteride pretreatment prevented thioacetamide-induced increases in acetylcholinesterase activity in the thalamus and caudate nucleus, with acetylcholinesterase activity showing a negative correlation in the thalamus (*p* < 0.05) and a positive correlation in the caudate nucleus (*p* < 0.01) (35). Although the causal relationship between finasteride and cognitive impairment remains unclear, the cholinergic system provides a plausible entry point for understanding its mechanism of action.

### 4.4 Hippocampus

The hippocampus is a brain region critically involved in learning and memory, as well as in behavioral responses to stress and the pathophysiology of mood disorders. These cognitive and emotional functions are closely interconnected. Hippocampal neurons and the hypothalamus form a dynamic interactive network that supports memory encoding, consolidation, and retrieval through direct neural circuits and indirect endocrine regulation (36). Giatti et al. (37) analyzed the gene expression profiles of hypothalamic and hippocampal tissues in adult male rats following finasteride intervention using RNA sequencing. Their findings revealed numerous differentially expressed genes closely associated with neurological emotion regulation, depressive-like behaviors, cognitive functions, and neurodegenerative processes (37). Furthermore, Römer et al. (38) evaluated the effects of finasteride on hippocampal neurogenesis in C57BL/6 N male mice using immunohistochemical methods. The study demonstrated that finasteride intervention significantly reduced brain levels of 5α-dihydrotestosterone, leading to a reversible decrease in the number of hippocampal newborn cells and immature neurons. However, the neurogenesis process returned to normal levels after 35 days of drug withdrawal (38).

These collective findings underscore the need for individualized therapeutic strategies considering patient-specific factors and risk–benefit profiles. Future research should elucidate population-specific effects, dose–response relationships, and duration-dependent cognitive impacts to optimize clinical decision-making.

### 4.5 Limitations

This study, while comprehensive, is subject to several limitations. First, the NHANES database is a cross-sectional study, primarily based on questionnaire responses, and provides observational data. The exclusion of certain individuals due to missing covariate data may introduce selection bias into the results. Additionally, this study employed subjective memory assessment methods to evaluate cognitive function, leaving out standardized cognitive impairment assessment scales, potentially introducing measurement bias. Furthermore, the inclusion of female participants in the study population means potential confounding factors, such as polycystic ovary syndrome (PCOS), which is known to induce cognitive impairment, could interfere with the interpretation of cognitive impairment-related outcomes. Second, the FAERS database relies on spontaneous reporting. This system is prone to omissions, misreporting, and incomplete information. Data is sourced from various stakeholders (e.g., pharmaceutical companies, patients, and healthcare providers), making reporting bias inevitable (39). Although this study combined the ROR method with the BCPNN method to refine the screening threshold for adverse drug event (ADE) signals, the potential for false-positive ADE signals cannot be entirely ruled out. Therefore, while this study demonstrates an association, it does not establish definitive causality. Therefore, further prospective studies are needed to corroborate these findings.

## 5 Conclusion

This study provides compelling evidence from both large-scale population-based data (NHANES) and real-world pharmacovigilance surveillance (FAERS) indicating a significant association between finasteride exposure and subjective memory impairment. Given this potential impact of finasteride on memory function, it is crucial to prioritize monitoring of cognitive status in clinical practice. Healthcare professionals should establish a comprehensive medication monitoring program, regularly assessing cognitive function—particularly memory—throughout the treatment period. This approach will ensure that any signs of cognitive impairment are promptly identified and addressed. Additionally, patients should be encouraged to adopt healthy lifestyle interventions, including a balanced diet, regular physical activity, sufficient sleep, and stress management strategies, all of which can help mitigate the risk of memory decline. Ultimately, tailored treatment and care plans should be developed based on individual differences, medication responses, and lifestyle factors to optimize overall health outcomes.

## Data Availability

The original contributions presented in the study are included in the article/supplementary material, further inquiries can be directed to the corresponding author.

## Footnotes

^1^ http://openvigil.sourceforge.net/
